# How Do Quality of Life (QoL) and Symptom Burden Evolve in Inpatient Palliative Care (PC) Patients following One Week of Care in a Specialized Palliative Care Unit (PCU)? A Comparison of Two Groups, with One Receiving Specialized Outpatient Palliative Care Prior to Admission

**DOI:** 10.3390/cancers16081612

**Published:** 2024-04-22

**Authors:** Hanna Salm, Florian Doberschütz, Franziska Hallmann, Philipp Munzert, Johannes Rahm, Sarah Uhlig, Daniel Pink

**Affiliations:** 1 Klinik und Poliklinik für Innere Medizin C, Universitätsmedizin Greifswald, 17489 Greifswald, Germanydaniel.pink@helios-gesundheit.de (D.P.); 2 Sarkomzentrum Berlin-Brandenburg, Helios Klinikum Bad Saarow, 15526 Bad Saarow, Germany

**Keywords:** specialized palliative care, outpatient palliative care, quality of life, symptom burden

## Abstract

**Simple Summary:**

The study aimed to assess changes in quality of life and symptom burden among palliative care patients during one week of inpatient care in a palliative care unit, comparing those who had prior outpatient palliative care with those who did not. Using the EORTC QLQ-C30 instrument, patients were evaluated at admission (T0) and one week later (T1). Patients in both groups showed significant and clinically meaningful improvements in global health/QoL, emotional functioning, nausea, vomiting, pain, and insomnia. The study suggests that even brief inpatient palliative care can substantially enhance the quality of life and reduce symptom burden, benefiting patients regardless of prior outpatient support.

**Abstract:**

Purpose: This study sought to investigate changes in quality of life (QoL) and symptom burden among palliative care patients undergoing one week of inpatient care in a specialized palliative care unit (PCU). The patient population was stratified into two groups, with one group pretreated from pre-admission palliative care (PC) provided by an outpatient multidisciplinary PC team, while the other group did not receive such support prior to admission. Although the average duration of treatment at a PCU in Germany is 1–2 weeks, the question also arises as to whether a significant improvement in symptom burden and QoL can be expected after just one week of PC in a PCU. Methods: PC patients with various cancer entities were prospectively included in a non-randomized study. Patients in group 1 received outpatient specialized PC prior to admission, while patients in group 2 did not. Over an 8-month period, we gathered data from one academic cancer center, utilizing the EORTC QLQ-C30, one of the most widely used patient-reported outcome (PRO) instruments to assess health-related QoL in cancer patients. Patients completed the QLQ-C30 at T0 (admission or one day later) and T1 (one week later), enabling the assessment of potential changes in their QoL and symptom burden over time. Results: A total of 103 patients (51.5% male) were enrolled (group 1: 42%, group 2: 58%). At T0, there were no significant differences regarding QLQ-C30 scores between groups 1 and 2, except from global health/QoL (group 1 mean 20.7, group 2 mean 25.6, *p* = 0.026). Over the course of one week several significant and clinically relevant changes were found: Emotional functioning demonstrated an uplift in both groups (group 1: mean 41.5 IQR 33 vs. 53.1 IQR 50, *p* = 0.014, group 2: mean 48.2 IQR 46 vs. 56.8 IQR 58, *p* = 0.029), as did the global health status (group 1: M 20.7 IQR 17 vs. 36.2 IQR 33, *p* < 0.001, group 2: M 25.6 IQR 25 vs. 35.3 IQR 33, *p* < 0.001). Nausea and vomiting showed a reduction (group 1: M 29.9 IQR 17 vs. 6.8 IQR 0, *p* < 0.001, group 2: M 22.6 IQR 17 vs. 8.2 IQR 0, *p* < 0.001), along with a notable decline in pain (group 1: M 67.4 IQR 67 vs. 25.3 IQR 17, *p* < 0.001, group 2: M 73.1 IQR 83 vs. 29.7 IQR 17, *p* < 0.001). A decrease was observed in insomnia (group 1: M 63.6 IQR 67 vs. 27.6 IQR 33, *p* < 0.001, group 2: M 60.1 IQR 67 vs. 27.6 IQR 33, *p* < 0.001). There were no significant differences between groups 1 and 2 in the extent of improvement in the various symptom scales from T0 to T1. Conclusion: The findings of our study demonstrate that QoL and several symptoms prevalent in cancer patients cared for in the PCU experienced significant enhancement over the span of just one week. Both groups, patients receiving specialized outpatient PC prior to admission and those without, equally benefited from inpatient PC. All mentioned changes from T0 to T1 are considered not only significant but clinically relevant.

## 1. Introduction

In the absence of a viable cure for a critically ill patient, the focus shifts from the pursuit of a cure to the management of symptoms and the improvement of QoL. Both aspects are the main goal of PC [1,2]. PC patients often experience numerous symptoms arising from both the treatment and the disease itself, such as fatigue, pain, lack of energy, appetite loss, and worry [3,4]. These symptoms interfere negatively with the QoL [5,6]. Addressing these symptoms typically involves the collaborative efforts of healthcare professionals from diverse disciplines within PC teams. The concept of PC includes psychological, social, and spiritual support, with the aim of achieving the highest possible QoL for patients and their families [7].

Thus, QoL evaluation is critically important for patients’ well-being. In the assessment of QoL and symptom burden, PRO instruments have become increasingly integral in clinical practice [8]. PRO measures are a valid method to determine patients’ own perceptions of their well-being, revealing a patient-centered view of their subjective experiences [9]. The utilization of PRO measures can facilitate improved decision-making for both patients and clinicians [10]. Thus, the recognition of the importance of incorporating patients’ views into clinical practice is widespread [11]. The EORTC QLQ-C30 health-related quality of life questionnaire stands out as one of the most widely utilized PRO instruments for evaluating health-related QoL in cancer patients [12]. 

The significance of PC enjoys broad acknowledgment, with both the World Health Organization (WHO) [7] and the American Society of Clinical Oncology (ASCO) strongly advocating for its provision. ASCO specifically recommends the early integration of PC into disease management [13], a stance supported by the findings of numerous studies [14,15,16,17]. Several studies have investigated the effects of inpatient PC on patients with cancer, indicating a range of benefits, such as decreased symptom severity [18,19] and increased disease awareness [20]. An admission to the palliative care unit can occur with any patient who has a life-limiting illness, currently suffers from a significant symptom burden, and where curative therapy is no longer the primary focus. The same reasons underlie the involvement of an outpatient PC team. A patient can be referred to an outpatient team through the clinic or their primary care physician. 

In addition to inpatient PC, specialized outpatient care has grown substantially. The aim is to sustain and enhance QoL and enable palliative patients to live in dignity until the end of life in their familiar surroundings [21]. Inpatient and outpatient care exhibit distinct differences in care delivery and process. Outpatient PC typically involves early referrals in the disease course, allowing for the enhancement of patient and family coping skills and a deeper understanding of the disease. Inpatient PC teams are often engaged later in the disease course, primarily focusing on acute symptom management and assisting in decision-making during hospitalization or clinical crises [22].

While studies have associated various benefits with both inpatient and outpatient PC, to our knowledge, there are no correlative studies comparing QoL at admission and changes in QoL and symptom burden after one week of inpatient PC between two groups, one of which received specialized outpatient PC prior to admission. It remains unexplored whether those patients derive similar advantages from inpatient PC. Thus, the objective of this study was to evaluate potential differences in the benefit of treatment in a specialized palliative care unit (PCU) between the groups. 

Additionally, we aimed to investigate whether a significant enhancement in QoL and symptom burden can be anticipated after just one week of treatment, considering the average duration of stay in PCU in Germany is typically 10 days. 

## 2. Methods

The study was a single-center, prospective study conducted at Helios Hospital Bad Saarow, Germany. Patients admitted to the PCU were recruited between March and October 2023. Patients were invited to complete the QLQ-C30 on the day of admission or the day after (T0) and one week later (T1). In addition, we collected data on clinical information. Prior to participation, patients received an information sheet detailing the study’s objectives. 

### 2.1. Patients

A total of 153 patients admitted to the PCU were invited to participate in the study. Following an initial discussion regarding the patient’s health status, a collective decision involving physicians and nurses was made to assess the patient’s eligibility for participation. If the patient’s health condition was deemed too severe, they were excluded from the study. To be eligible for inclusion, patients had to have been diagnosed with a severe illness, predominantly cancer, in this study, be over 18 years old, and possess the ability to communicate effectively with the staff. Patients with cognitive impairment were excluded from the study. 

Patients admitted to the PCU were those unable to receive treatment at home due to symptom burden or social issues. They received daily follow-up care from physicians and nurses, with additional support from other staff members such as physiotherapists, psychologists, social workers, and volunteers, depending on patients’ needs. 

Patients who were cared for at home by a specialized outpatient PC team prior to admission to the PCU were visited by nurses once a day and by a physician at least once a week. Patients receiving care at home did not have access to all members of the multidisciplinary team such as later in the hospital. 

### 2.2. Outcome Measure

The QLQ-C30 is a 30-item questionnaire covering five functional scales (physical, role, cognitive, social, and emotional functioning), three symptom scales (fatigue, nausea/vomiting, and pain), five single-item symptom scales (dyspnea, insomnia, appetite loss, constipation, and diarrhea), and a single-item scale for financial impact due to the disease and treatment. It also includes a global health/quality of life (QoL) scale composed of two items. Higher scores on functional scales indicate better functioning, while higher scores on symptom scales indicate greater symptom burden. Previous studies show its reliability and validity in measuring QoL in PC [23].

### 2.3. Statistical Analysis

Absolute and percentage frequencies were used to describe qualitative variables. Quantitative variables were described using either the mean with standard deviation or the median with range, depending on the distribution. The Kolmogorov–Smirnov test showed that the EORTC scale values exhibited significant deviations from a normal distribution so that the comparison of the two patient groups with vs. without SAP care was carried out using the non-parametric Mann–Whitney U test and the Wilcoxon Signed Ranks test was used to test whether the patients in the two groups changed significantly in the EORTC scales between the two data collection times before vs. after the intervention. 

All statistical tests were two-sided, and a significance level of 5% was used. There was no alpha adjustment for multiple testing, so the results are exploratory and descriptive in nature. IBM SPSS Statistics 28 (SPSS Inc., an IBM Company, Chicago, IL, USA) was used to perform the statistical calculations.

### 2.4. Compliance with Ethical Standards

The study protocol received approval from the Ethics Committee of the University of Greifswald, as well as from the Medical Association of Brandenburg and the Ethics Committee of the Helios Hospital Bad Saarow. Written informed consent was obtained from all patients, and the study was conducted in accordance with the Declaration of Helsinki.

## 3. Results

### 3.1. Participation

**Figure 1:**
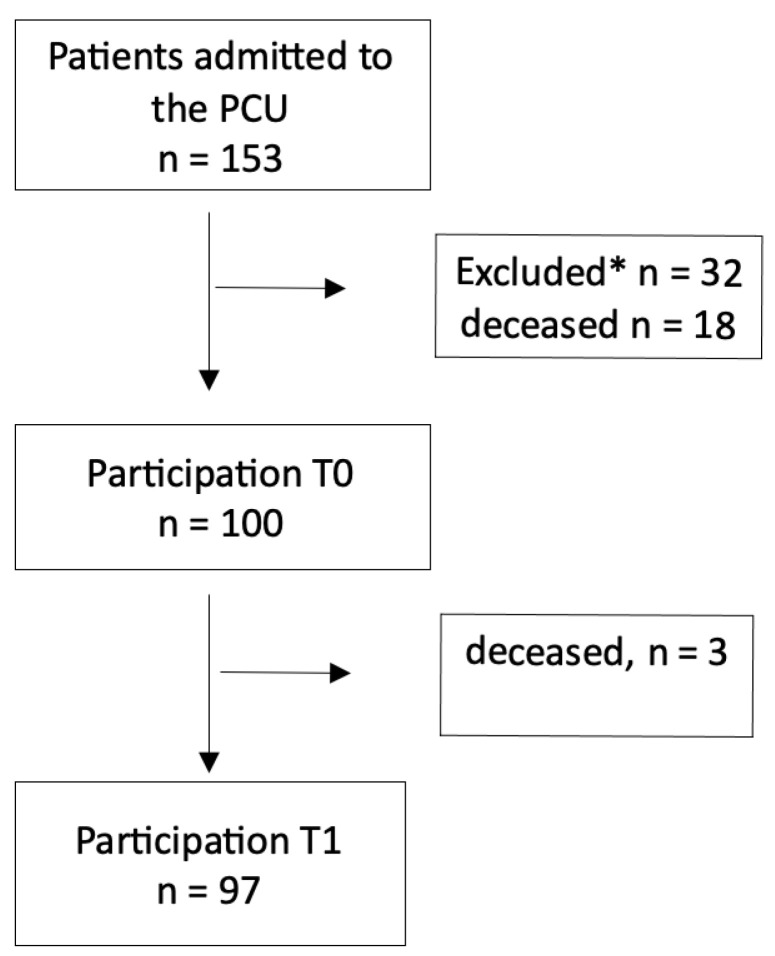
Study participation. * Reasons for exclusion: language barrier (1), Patient refusal (1), transfer or discharge (3), too ill (27).

During an eight-month study period, a total of 153 patients were admitted to the PCU as shown in Figure 1. Among them, 27 patients were considered too ill to participate, while one patient encountered a language barrier, and another refused to participate. Additionally, three patients were transferred or discharged before the study commenced, and 18 patients deceased before study initiation. Ultimately, 103 patients participated in the study, with three more patients passing away before T1.

### 3.2. Study Population

A total of 103 patients participated at T0. Fifty-three patients (48.5%) were male and 50 (51.5%) were female. Forty-three patients (42%) were pretreated by a specialized outpatient PC team prior to admission (group 1), while 60 patients (58%) received no outpatient PC prior to admission (group 2). Patients in group 1 were aged from 35 and 87 (mean 72.4, SD 11.3), and patients in group 2 ranged in age from 48 and 87 (mean 72.0, SD 9.4). Malignancy was the main reason for palliative consult; four patients (3.8%) had no underlying malignancy. Demographic data are presented in Table 1.

At T0, there were no significant differences regarding symptom burden between groups 1 and 2. However, group 2 exhibited a higher score in global health/QoL at the time of admission (group 1 mean 20.7, group 2 mean 25.6, *p* = 0.026). 

### 3.3. Changes in QoL and Symptom Burden from T0 to T1

Over the course of one week, several significant changes in QoL and symptom burden were observed: Emotional functioning demonstrated improvement in both groups (group 1: mean 41.5 IQR 33 vs. 53.1 IQR 50, *p* = 0.014, group 2: mean 48.2 IQR 46 vs. 56.8 IQR 58, *p* = 0.029), as did the global health status (group 1: mean 20.7 IQR 17 vs. 36.2 IQR 33, *p* < 0.001, group 2: mean 25.6 IQR 25 vs. 35.3 IQR 33, *p* < 0.001). Nausea and vomiting showed a reduction (group 1: mean 29.9 IQR 17 vs. 6.8 IQR 0, *p* < 0.001, group 2: mean 22.6 IQR 17 vs. 8.2 IQR 0, *p* < 0.001), along with a notable decline in pain (group 1: M 67.4 IQR 67 vs. 25.3 IQR 17, *p* < 0.001, group 2: mean 73.1 IQR 83 vs. 29.7 IQR 17, *p* < 0.001). A decrease was observed in insomnia (group 1: mean 63.6 IQR 67 vs. 27.6 IQR 33, *p* < 0.001, group 2: mean 60.1 IQR 67 vs. 27.6 IQR 33, *p* < 0.001). All improvements with a mean change of at least 10 points [24,25] were considered not only significant but also clinically relevant. With this interpretation in the QLQ-C30, emotional functioning as well as social functioning showed a clinically relevant uplift in group 1, as did the global health status/QoL in both groups. A clinically relevant decrease was shown in nausea and vomiting, pain, insomnia, appetite loss, and constipation in both groups. Diarrhea decreased clinically relevant in group 2. 

Additionally, some domains of QoL did not show significant enhancement during the study. Specifically, physical functioning did not demonstrate improvement in either group (group 1: mean 16, IQR 7.0 vs. 15.2, IQR 7.0, *p* = 0.781, group 2: mean 18.7 IQR 13.0 vs. 13.8 IQR 7.0, *p* = 0.045). Cognitive functioning also did not change significantly in either group (group 1: mean 57.7, IQR 50 vs. 64.2, IQR 67.0, *p* = 0.201; group 2: mean 66.1, IQR 67.0 vs. 58.8, IQR 67.0, *p* = 0.115). Similarly, dyspnea did not alleviate in either group (group 1: mean 41.8, IQR 33.0 vs. 34.8, IQR 33.0, *p* = 0.436; group 2: mean 46.5, IQR 33.0 vs. 37.2, IQR 33.0, *p* = 0.080). Furthermore, in group 2, diarrhea did not show a significant decrease (mean 17.1, IQR 0.0 vs. 8.1, IQR 0.0, *p* = 0.164).

There were no significant differences between groups 1 and 2 in the extent of improvement in the various symptom scales from T0 to T1. However, it is worth noting that certain symptoms showed a more pronounced improvement in one of the groups, as shown in Table 2.

## 4. Discussion

### 4.1. Summary of the Findings

The study’s primary objective was to investigate potential disparities in QoL and symptom burden following one week of treatment in a PCU between patients who had previously received care from a specialized outpatient PC team and those who had not prior to admission. In the study planning phase, we anticipated that patients with prior outpatient palliative care might show a greater symptom burden upon admission and that achieving symptom improvement through inpatient palliative treatment could be more difficult for these individuals. However, our results did not confirm these assumptions; instead, both groups demonstrated similar benefits.

Upon admission, no significant differences in symptom burden were observed between the groups, irrespective of whether they had been accompanied by an outpatient PC team before admission or not. Remarkably, group 2, which had not availed of outpatient PC care prior to admission, exhibited a higher score in global health/QoL at admission. This finding may suggest that group 2 did not require outpatient PC due to their generally higher well-being. 

Our findings revealed a significant enhancement in several symptoms and functional status within both groups during their stay at the PCU. Significant improvements were not only considered significant but also clinically relevant, demonstrating a change of at least 10 points [24,25]. However, no discernible differences were observed between the groups. This suggests that patients, irrespective of whether they received PC before admission or not, experienced similar benefits from one week of inpatient PC. 

Furthermore, our results underline the efficacy of PC, with significant enhancements evident after just one week, despite the typical duration of stay in a palliative unit in Germany being 10 days.

We want to emphasize that the absence of differences in admission conditions and post-intervention outcomes between the two groups does not mean that outpatient PC could be useless. The results do not question the effectiveness of outpatient PC, as this study does not tell us how many patients avoided hospital admission because they received early outpatient PC.

### 4.2. Change in QoL and Symptom Burden

PC exposure was associated with a significant improvement in QoL across both groups. We were able to illustrate a distinct enhancement in symptom control, which is in line with previous studies [25]: nearly all QLQ-C30 symptom scales (pain) and single items (fatigue, nausea and vomiting, insomnia, appetite loss, and constipation) demonstrated significant improvement. However, dyspnea did not exhibit improvement in either group, suggesting potential challenges in managing dyspnea, particularly in the terminal stages of life. 

Both groups showed improvement in emotional functioning, which could be linked to the psychological support offered to all patients. Psychological support holds particular importance in PC, given that mood disorders, such as depression, can coexist at a frequency of about 25% in patients with advanced cancer [26,27]. Terminally ill patients with depression may still derive benefits from treatment, even during the final weeks of life [28,29].

Physical functioning improved in group 2 but not in group 1. This might be explained by the advanced stage of cancer and its progression [30], which could be more prevalent in patients already receiving pretreatment from an outpatient PC team due to their compromised functional status. Cognitive functioning exhibited no significant change over the span of one week, which may have been associated with disease progression and the use of medications, especially opioids [31]. Global health status in both groups improved significantly, even though, according to Elmqvist et al., the QoL of advanced cancer patients decreased in the last three months of life [30]. However, we lack information regarding the remaining lifespan of patients after completing the study.

Although most symptoms improved following inpatient PC exposure, certain aspects of QoL did not exhibit significant enhancement. Specifically, physical functioning did not show improvement in either of the groups, which is challenging to explain given that patients routinely received physiotherapy. In the context of declining health, physiotherapy is viewed as a potential means to enhance QoL through passive movement or relaxation, but it may not always lead to substantial changes in mobility. Moreover, cognitive functioning and severity did not improve in either group. This could be attributed to the severity of the disease or possibly the need for a longer duration of stay to adequately address these symptoms. Additionally, non-medical factors such as emotional and social aspects may have influenced these symptoms, highlighting the inherent complexity of symptom management. Furthermore, diarrhea did not demonstrate a significant decrease in group 2. This may be due to group 2 receiving prior treatment from an outpatient PC team, which could have addressed such issues preemptively.

In certain aspects, notable differences were observed between the groups. It remains unclear why patients in group 2 experienced improvements in diarrhea and financial difficulties while those in group 1 did not. Similarly, it is unclear why group 1 showed improvement in social functioning, whereas group 2 did not. Given the challenges in pinpointing a definitive explanation, it is conceivable that these discrepancies may have arisen by chance. Further investigation and analysis are warranted to elucidate these findings.

## 5. Strength and Limitation

To the best of our knowledge, this study represents the first investigation into the effects of treatment at a PCU, comparing two groups, one of which had received prior treatment from an outpatient specialized PC team. Some limitations of the study should be acknowledged, including the recruitment of patients from a single institution and the modest sample size, which encompassed diverse tumor entities. 

QoL assessment was conducted within a short timeframe of 7 days, without follow-up, and a considerable number of patients were unable to participate due to severe illness or death before the study commenced. It is conceivable that the symptoms could have shown even greater improvement if T1 had taken place at a later time instead of after one week. We chose not to schedule T1 at discharge to ensure that all patients received care for the same duration, facilitating more accurate comparisons of changes over time. This timeframe also aligns with common practices in Germany, where many patients typically receive approximately one week of inpatient palliative care. Our objective was to evaluate both baseline (T0) and early treatment effects (T1) for as many study participants as possible. It is noteworthy that we observed substantial and statistically significant improvements after just one week.

Further on, we were unable to assess the duration of outpatient treatment prior to admission received by patients in group 1. It is conceivable that variations in the duration of outpatient PC may have influenced the extent of benefits observed during their stay at a PCU. 

Additionally, there were variations between the groups at T0; notably, group 2 exhibited a higher score in global health/QoL at the time of admission. It is crucial to acknowledge that this difference limited the comparability of the groups upon admission.

## 6. Conclusions

In conclusion, QoL improved in both patient groups, comparing baseline and follow-up assessments after one week. Our findings suggest that patients, regardless of prior exposure to outpatient PC, derived similar benefits from care at a PCU. The symptom burden of both groups was similar upon admission, and the improvement in symptom burden after one week was not only statistically significant but also clinically relevant. Thus, our results underscore the importance of considering inpatient PC as a valuable intervention, irrespective of previous outpatient PC received. 

Studies with larger sample sizes are needed to confirm our results and to allow their generalization. 

## Figures and Tables

**Table 1:** Demographics and clinical characteristics of the study population.

Variable	Value	Group 1 (n; %)	Group 2 (n; %)
Sex	female	24 (55.8)	26 (43.3)
	male	19 (44.2)	34 (56.7)
Age		72.4, 11.3 (m, SD)	72.0, 0.4 (m, SD)
	head/neck	11 (25.6)	7 (11.7)
	lung	8 (18.6)	13 (21.7)
	colon	7 (16.2)	9 (15.0)
	breast	4 (9.3)	3 (5.0)
	pancreas	2 (4.7)	4 (6.7)
	others	7 (16.3)	17 (28.2)
	hematological neoplasia	3 (7.0)	4 (6.7)
	no malignancy	1 (2.3)	3 (5.0)

**Table 2:** Change in QoL and symptom burden from T0 to T1. Bold format: significant result.

	Group 1 (Ambulant Palliative Care Prior to Admission)							Group 2 (No Palliative Care Prior to Admission)						
	T0			T1				T0			T1			
	Mean	SD	IQR	Mean	SD	IQR	*p*	Mean	SD	IQR	Mean	SD	IQR	*p*
Physical functioning	16.0	21.2	7.0	15.2	19.3	7.0	0.781	18.7	21.2	13.0	13.8	17.2	7.0	**0.045**
Role functioning	10.1	19.8	0.0	15.6	23.6	0.0	**0.048**	16.3	23.4	0.0	11.6	19.5	0.0	0.311
Emotional functioning	41.5	26.5	33.0	53.1	22.9	50.0	**0.014**	48.2	24.2	46.0	56.8	27.6	58.0	**0.029**
Cognitive functioning	57.7	30.5	50.0	64.2	33.7	67.0	0.201	66.1	27.4	67.0	58.8	32.5	67.0	0.115
Social functioning	37.6	30.3	33.0	50.1	31.2	50.0	**0.013**	42.8	32.4	50.0	48.3	29.7	50.0	0.206
Global health status/QoL	20.7	11.0	17.0	36.2	20.8	33.0	**<0.001**	25.6	13.7	25.0	35.3	21.2	33.0	**<0.001**
Fatigue	88.2	17.4	100.0	80.0	23.3	89.0	**0.016**	87.2	19.6	100.0	80.5	21.0	89.0	**0.004**
Nausea and vomiting	29.9	35.4	17.0	6.8	17.6	0.0	**<0.001**	22.6	29.9	17.0	8.2	19.9	0.0	**<0.001**
Pain	67.4	34.5	67.0	25.3	21.4	17.0	**<0.001**	73.1	31.6	83.0	29.7	24.1	17.0	**<0.001**
Dyspnea	41.8	35.8	33.0	34.8	37.3	33.0	0.436	46.5	36.5	33.0	37.2	35.0	33.0	0.080
Insomnia	63.6	37.0	67.0	27.6	28.8	33.0	**<0.001**	60.1	33.6	67.0	27.6	31.1	33.0	**<0.001**
Appetite loss	78.3	29.8	100.0	54.5	37.9	67.0	**0.001**	76.6	30.3	100.0	58.2	36.0	67.0	**<0.001**
Constipation	49.5	43.8	67.0	9.7	20.0	0.0	**<0.001**	48.3	41.0	33.0	18.6	27.9	0.0	**<0.001**
Diarrhoe	17.1	35.8	0.0	8.1	19.4	0.0	0.164	12.8	27.5	0.0	2.7	11.1	0.0	**0.012**
Financial difficulties	8.5	20.8	0.0	6.5	17.0	0.0	0.713	6.6	16.0	0.0	2.3	10.5	0.0	**0.020**

## Data Availability

The data can be shared up on request.
